# Supplementary material for: Phylotranscriptomics unveil a Paleoproterozoic-Mesoproterozoic origin and deep relationships of the Viridiplantae
Source: Nat Commun. 2023 Sep 11;14:5542. doi: 10.1038/s41467-023-41137-5 (PMC10495350; doi:10.1038/s41467-023-41137-5)
Supplement: Supplementary file 1 — Supplementary Information [file 41467_2023_41137_MOESM1_ESM.pdf]

Supplementary Information for

Phylotranscriptomics unveil a Paleoproterozoic-Mesoproterozoic origin and deep relationships of the Viridiplantae

Zhiping Yang[1], Xiaoya Ma[1], Qiuping Wang[1], Xiaolin Tian[1], Jingyan Sun[1], Zhenghua Zhang[1], Shuhai Xiao[2], Olivier De Clerck[3], Frederik Leliaert[4], Bojian Zhong[1,*]

Corresponding author. Bojian Zhong, College of Life Sciences, Nanjing Normal University, Nanjing, China, email: bjzhong@gmail.com

This PDF file includes:

Supplementary Figures 1-14

Supplementary Tables 1-2

Supplementary References

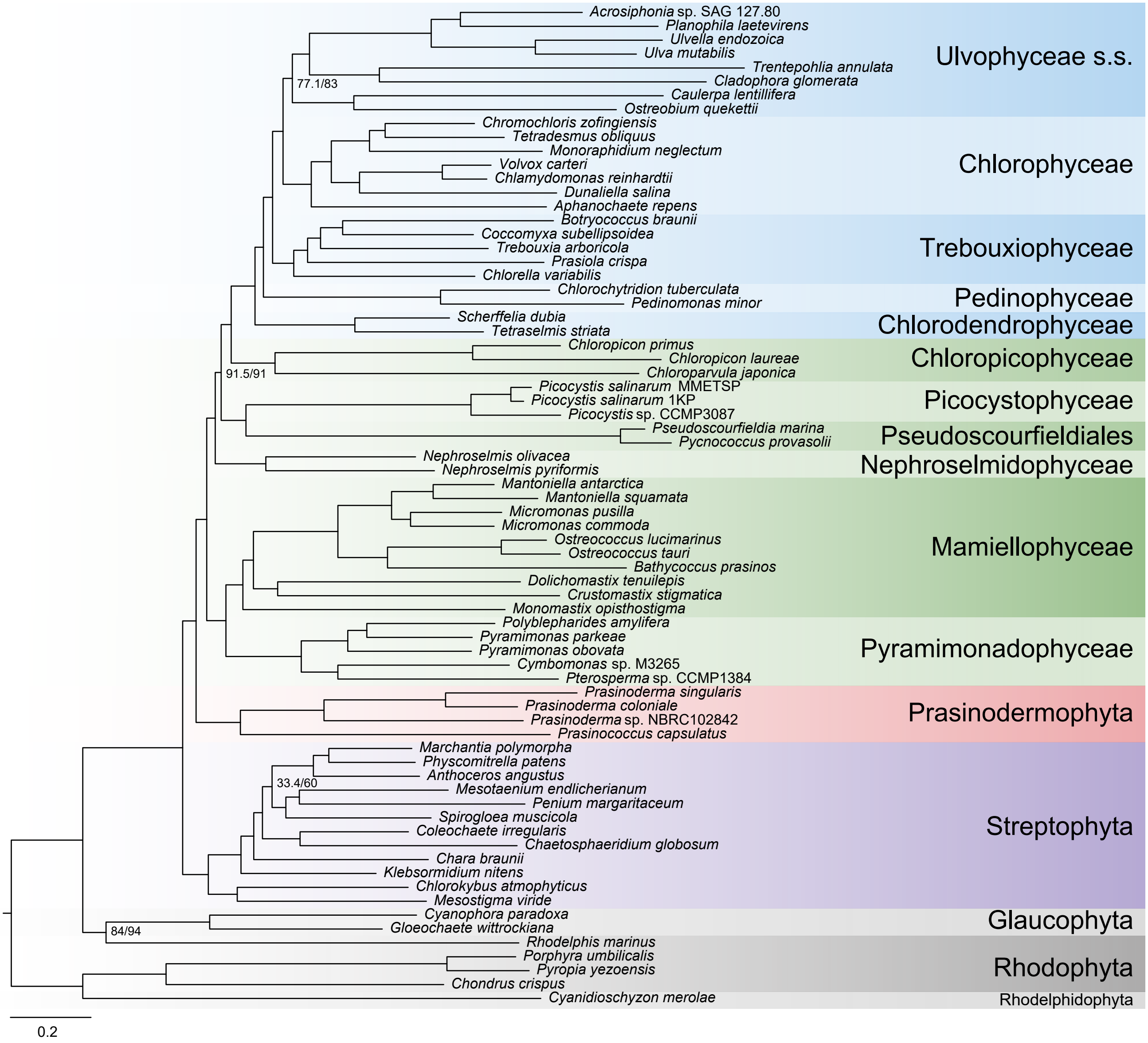

Supplementary Fig. 1 Phylogenetic trees reconstructed using the maximum-likelihood method in IQ-TREE based on a concatenation analysis of 557 SCOGs under the site-heterogeneous LG+C20+F+G model. Support values are shown only for nodes receiving less than 95% support from SH-aLRT/BS analyses.

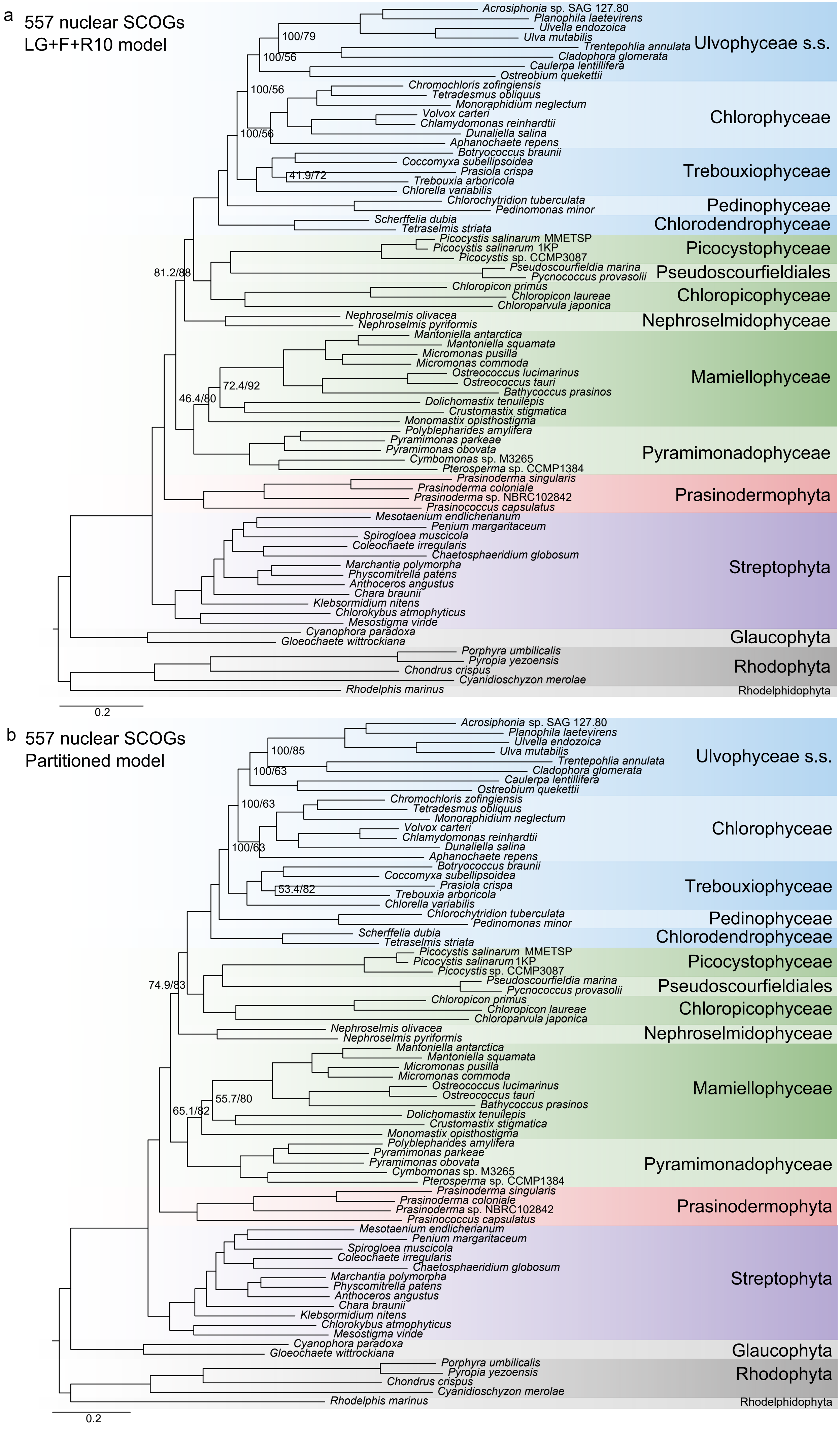

Supplementary Fig. 2 Phylogenetic trees reconstructed using the maximum-likelihood method in IQ-TREE based on a concatenation analysis of 557 SCOGs under (a) the site-homogeneous LG+F+R10 model and (b) the site-homogeneous model (partitioned by gene). Support values are shown only for nodes receiving less than 95% support from SH-aLRT/BS analyses.

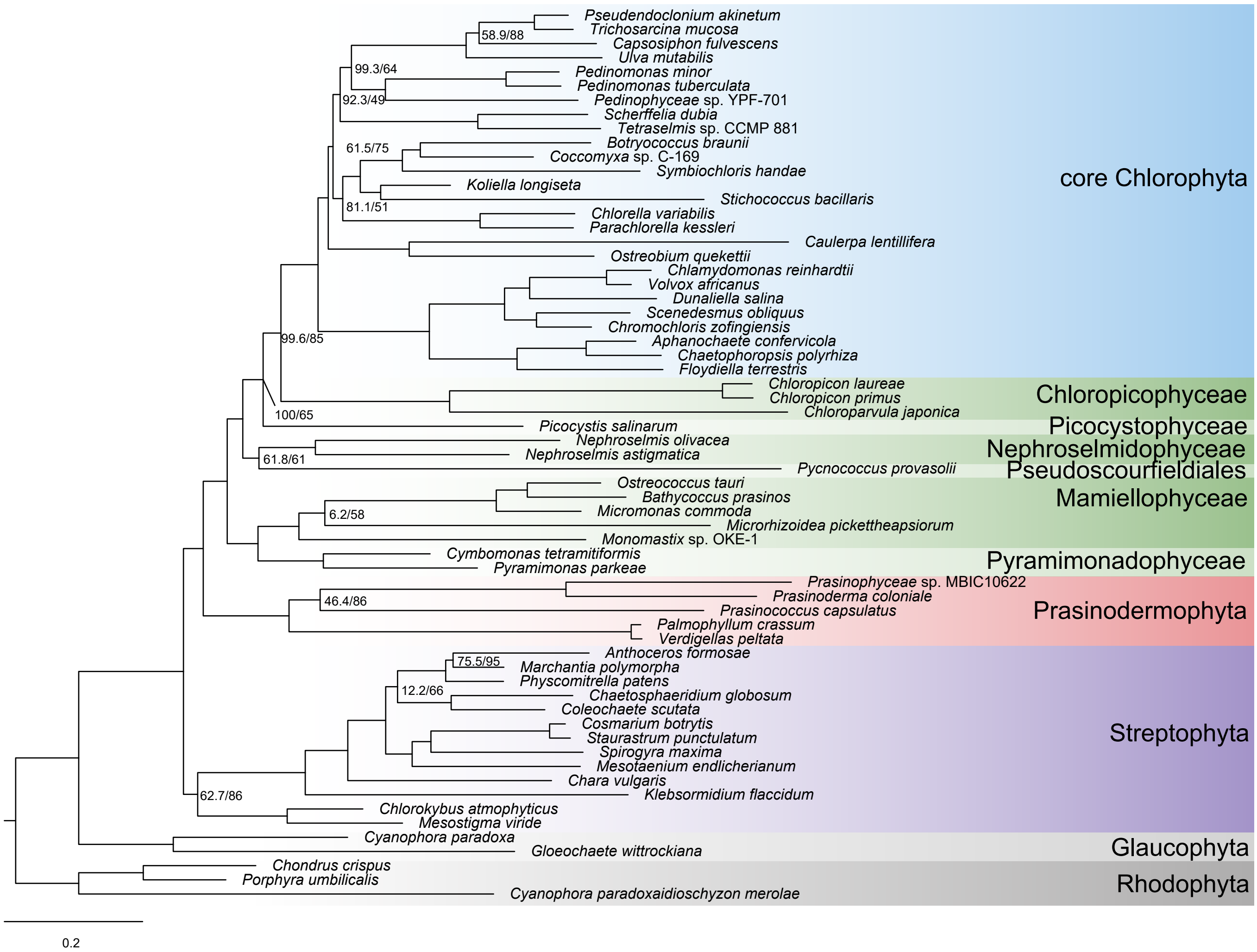

Supplementary Fig. 3 The plastid phylogenies of early-diverging green plants obtained by maximum-likelihood inference (IQ-TREE) of a concatenated alignment of 74 genes using the site-heterogeneous cpREV+C20+F+G model. Support values are shown only for nodes receiving less than 95% support from SH-aLRT/BS analyses.

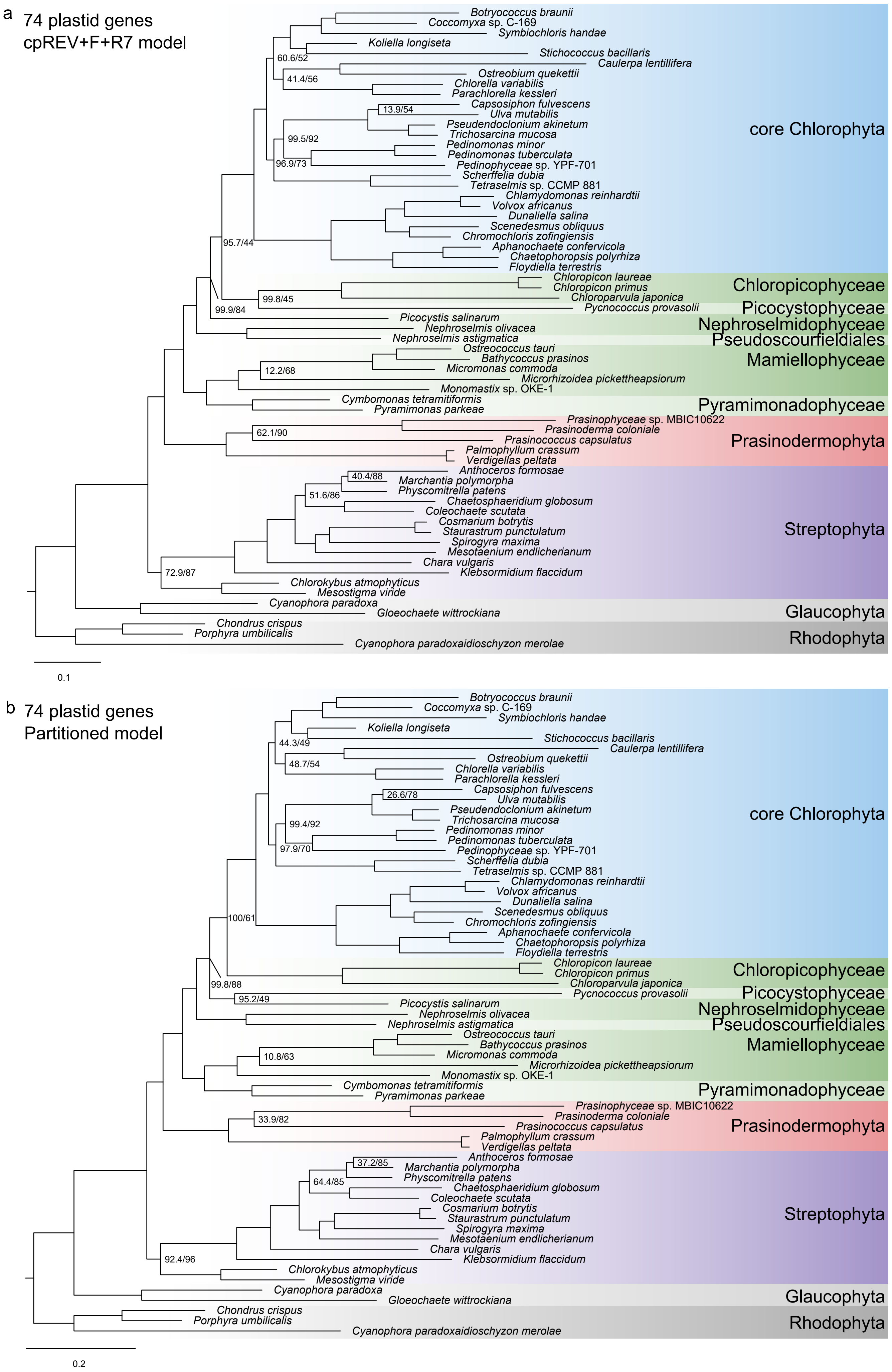

Supplementary Fig.4 The plastid phylogeny of early-diverging green plants obtained by maximum-likelihood inference (IQ-TREE) of a concatenated alignment of 74 genes using (a) the site-homogeneous cpREV+F+R7 model and (b) gene-wise partitioned strategy. Support values are shown only for nodes receiving less than 95% support from SH-aLRT/BS analyses.

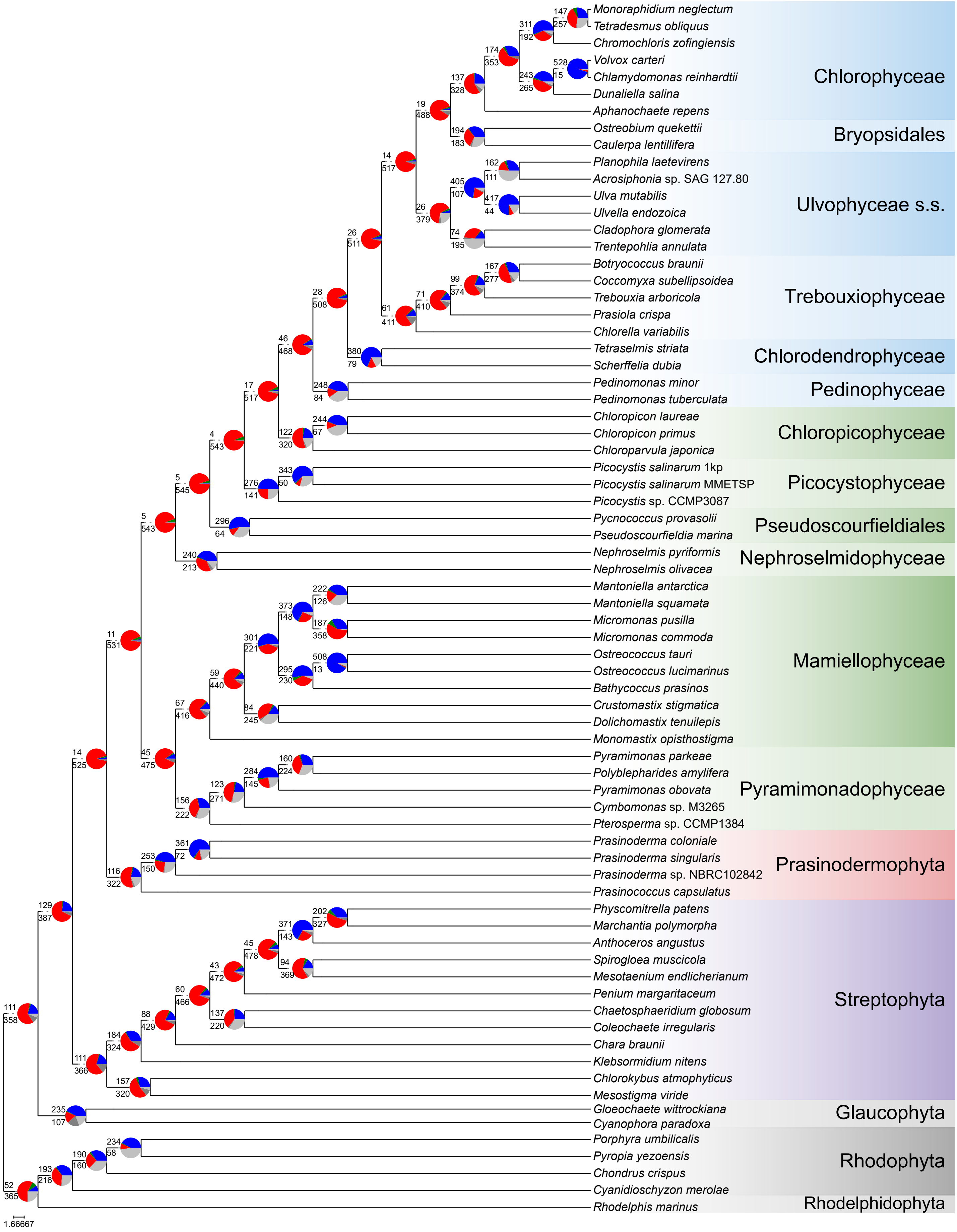

Supplementary Fig. 5 The coalescent species tree of 557 nuclear genes with summary of gene tree concordance and conflict. Numbers above or below branches indicate the number of gene trees concordant or conflicting with that branch in the species tree. Pie chart color coding: blue, the proportion of gene trees supporting the species tree; green, the proportion of gene trees supporting the most common conflicting topology; red, the proportion of gene trees supporting all other conflicting topologies; dark gray, the proportion of the uninformative gene trees; light gray, the proportion of missing data.

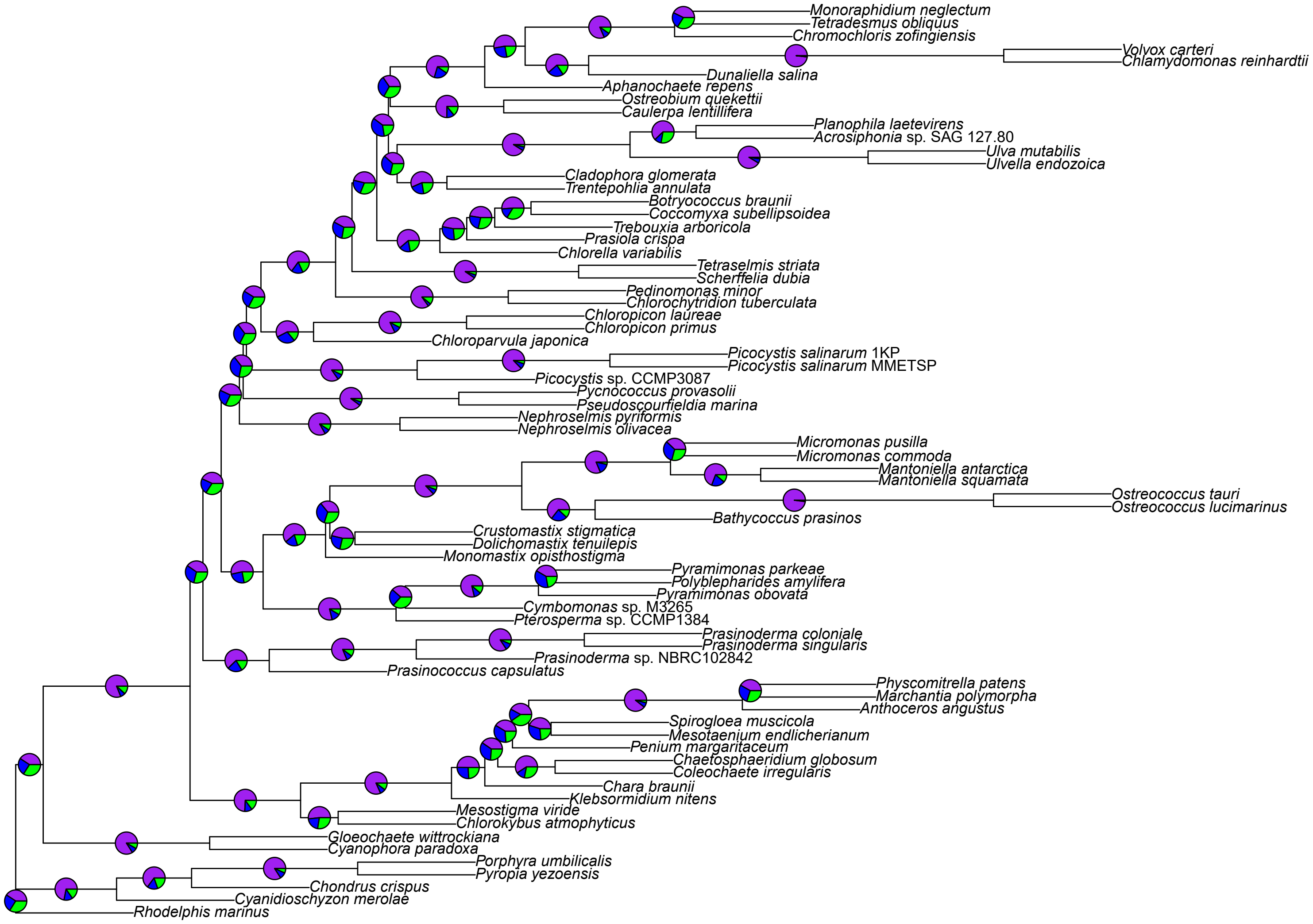

Supplementary Fig. 6 The pie chart on each internal branch of ASTRAL tree represents the quartet frequencies surrounding that branch calculated from 557 individual gene trees. Purple, the proportion of quartet 1 (q1) generated from '-t 8' in ASTRAL; Blue, the proportion of quartet 2 (q2); Green, the proportion of quartet 3 (q3).

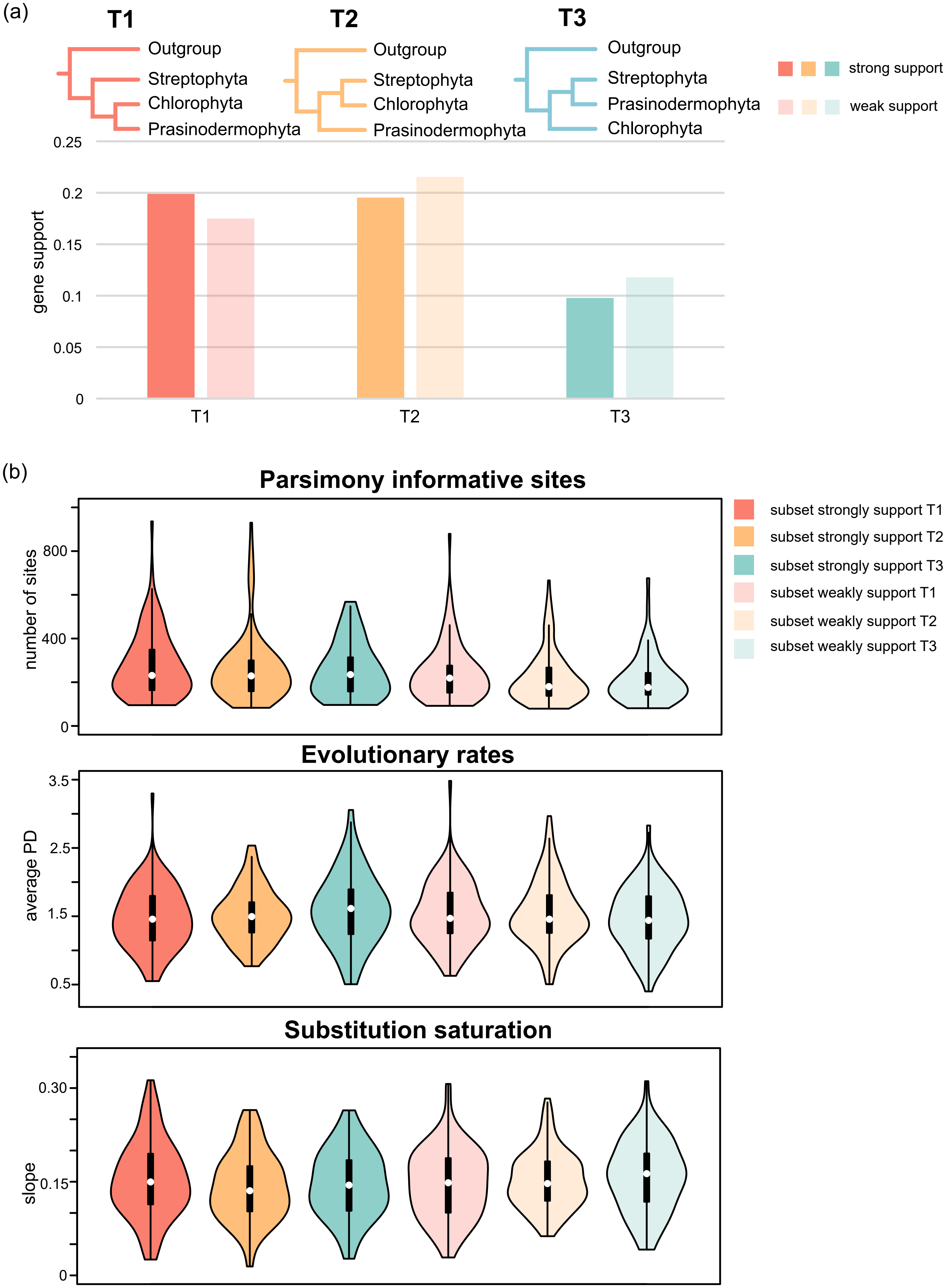

Supplementary Fig. 7 (a) The distribution of phylogenetic signal for three hypotheses concerning the position of Prasinodermophyta. The columns represent the proportion of genes with strong or weak signal supporting T1, T2, and T3 in 557 SCOGs. (b) Dissection of phylogenetic signal in terms of parsimony informative sites, saturation, and evolutionary rates. The more the number of informative sites, the more gene signal. The higher the slope the less saturated is the gene. The higher average patristic distance (PD) the faster evolutionary rate is the gene. The white dot represents the median and the black bar in the center of a violin plot represents the interquartile range.

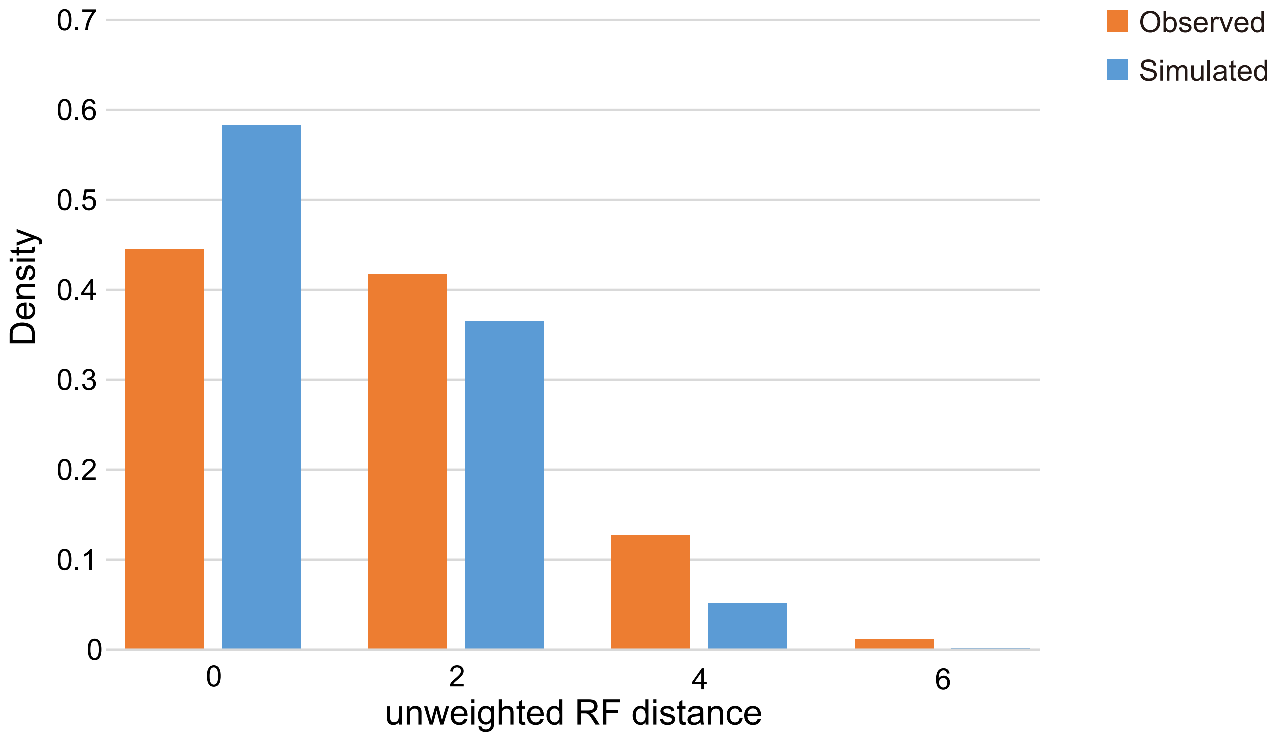

Supplementary Fig. 8 Histograms of the observed and simulated RF distances based on the 7-taxon dataset. The average of the observed RF distance is 1.41, whereas the average of the simulated RF distance is 0.94. The ratio 0.94/1.41= 66% is used to measure the proportion of gene tree variation (i.e., distance) that can be explained by the coalescent model.

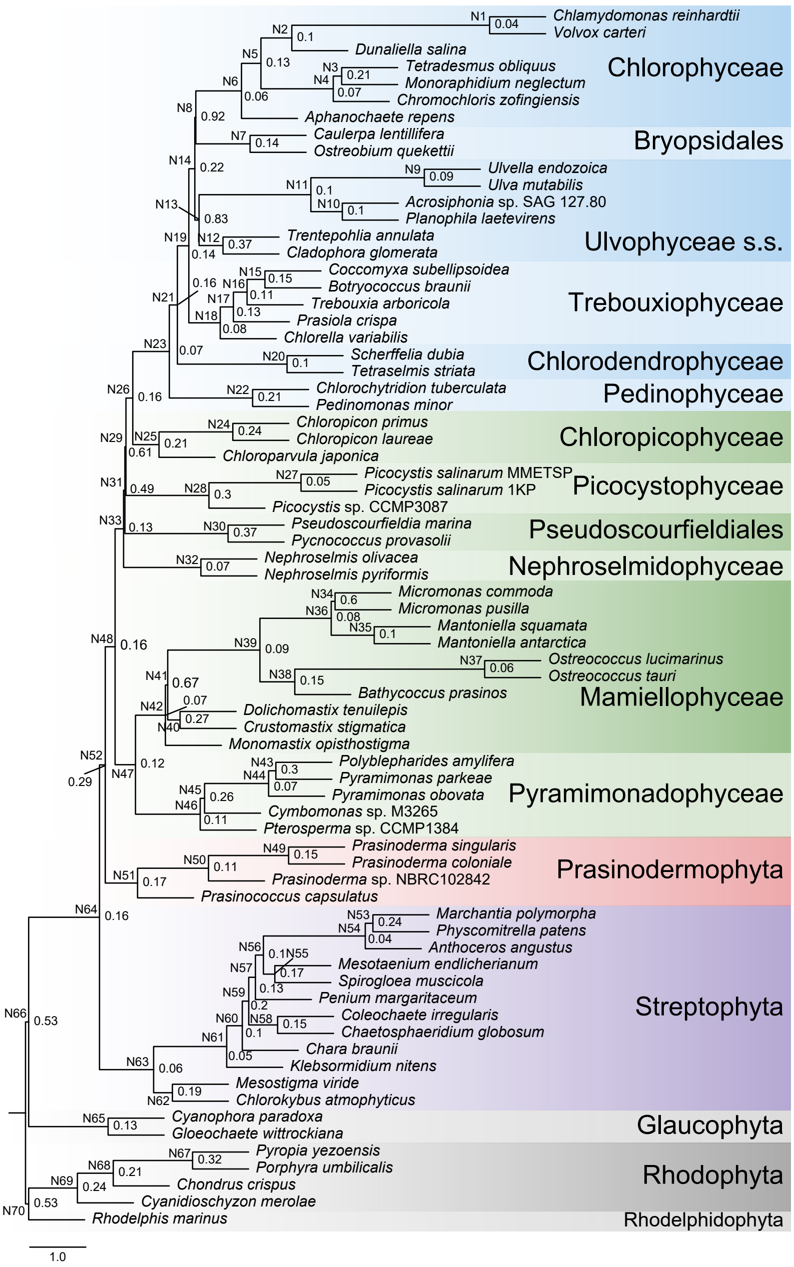

Internal branch ID	Frequence of minor quartet 1		Frequence of minor quartet 2		$\chi 2$	*P* value	QD value
	Observed	Simulated	Observed	Simulated			
N1	3.10	6.00	5.87	4.00	1.227	0.268	1.00
N2	68.62	2211.23	105.49	2194.59	7.783	0.005	0.29
N3	88.16	4922.96	128.66	4996.94	6.825	0.009	0.50
N4	45.60	1075.98	41.12	1004.76	0.026	0.873	0.62
N5	92.84	3358.20	84.53	3278.89	0.211	0.646	0.84
N6	30.13	1296.93	63.51	1296.01	11.509	0.001	0.90
N7	34.42	6640.68	44.75	6642.16	1.337	0.248	1.00
N8	95.52	6392.55	95.25	6446.23	0.006	0.939	0.97
N9	28.81	107.00	9.18	115.50	10.006	0.002	0.89
N10	26.71	121.00	74.54	118.00	17.019	3.70E-05	0.48
N11	20.56	114.81	12.37	119.83	2.110	0.146	0.67
N12	42.60	5537.80	46.10	5492.22	0.167	0.683	0.86
N13	68.78	6689.45	78.81	6637.20	0.755	0.385	0.71
N14	62.61	5350.19	105.73	5453.67	10.079	0.001	0.82
N15	51.68	3525.19	117.27	3473.71	25.816	3.76E-07	0.54
N16	96.99	4142.90	81.81	4091.99	1.084	0.298	0.76
N17	74.80	4113.72	94.43	4115.33	2.222	0.136	0.80
N18	63.97	816.37	49.52	807.32	1.572	0.210	0.88
N19	88.71	4506.78	64.61	4352.97	2.948	0.086	0.90
N20	19.54	18.71	19.91	16.88	0.069	0.792	0.86
N21	75.50	4987.08	79.64	5008.88	0.092	0.762	0.89
N22	13.46	445.46	32.48	436.69	7.849	0.005	0.67
N23	49.26	1833.16	47.16	1742.45	0.001	0.972	0.82
N24	25.78	448.33	20.41	478.70	0.975	0.323	0.42
N25	42.09	4484.47	82.59	4527.89	12.590	3.88E-04	0.77
N26	91.94	5304.44	71.75	5212.17	2.116	0.146	0.67
N27	27.49	221.00	16.63	249.00	3.773	0.052	1.00
N28	25.69	385.36	28.66	352.97	0.492	0.483	0.91
N29	86.94	6434.20	80.84	6458.99	0.243	0.622	0.91
N30	18.28	418.40	17.07	436.02	0.102	0.749	0.80
N31	66.07	6268.41	85.54	6383.52	2.133	0.144	0.80
N32	26.25	1.54	31.34	3.68	0.503	0.478	0.50
N33	75.57	4895.52	57.71	4889.82	2.339	0.126	0.67
N34	169.63	5855.48	150.52	5800.51	0.942	0.332	0.91
N35	68.20	6128.00	38.49	6132.46	8.224	0.004	1.00
N36	16.87	551.50	76.13	525.83	37.432	9.46E-10	0.40
N37	7.26	9.78	3.30	5.17	0.032	0.859	–
N38	61.17	6155.00	119.53	6090.53	19.184	1.19E-05	0.50
N39	24.61	218.73	32.19	244.87	0.301	0.583	0.50
N40	65.34	5007.32	76.55	5168.17	0.558	0.455	0.61
N41	87.47	6139.65	98.47	6117.03	0.682	0.409	0.75
N42	55.88	2281.04	50.53	2327.83	0.380	0.537	0.89
N43	124.06	5146.00	76.27	5165.03	11.354	0.001	0.63
N44	34.14	682.54	32.12	680.35	0.052	0.819	0.62
N45	92.36	5738.00	63.01	5681.35	5.188	0.023	0.74
N46	22.36	500.60	34.06	522.95	1.842	0.175	0.33
N47	55.43	2095.73	62.99	2124.81	0.374	0.541	0.90
N48	87.93	4707.03	58.21	4716.71	6.015	0.014	0.72
N49	32.83	400.00	21.64	387.00	1.820	0.177	0.73
N50	31.38	531.55	25.86	549.90	0.699	0.403	1.00
N51	46.66	5004.83	60.99	4974.65	1.974	0.160	0.69
N52	82.61	5429.18	88.44	5482.97	0.143	0.705	0.97
N53	143.13	5011.00	154.32	5177.00	0.131	0.717	0.78
N54	18.01	139.93	34.42	156.15	2.997	0.083	0.86
N55	124.55	6593.54	92.09	6720.43	5.416	0.020	1.00
N56	155.19	5028.56	63.48	5039.01	37.835	7.70E-10	0.45
N57	85.02	5331.96	125.05	5273.15	7.921	0.005	0.94
N58	38.54	1693.12	82.55	1629.48	17.101	3.54E-05	0.67
N59	103.90	5302.78	131.36	5277.41	3.265	0.071	0.79
N60	99.87	3807.40	107.93	3835.08	0.250	0.617	0.64
N61	43.26	499.17	32.85	513.28	1.606	0.205	0.83
N62	100.70	3054.20	73.85	2926.80	2.979	0.084	0.92
N63	49.41	2.25	34.59	1.86	0.027	0.869	1.00
N64	47.47	555.71	29.09	486.09	2.154	0.142	0.73
N65	33.26	5.00	34.27	2.00	1.249	0.264	0.71
N66	82.91	6027.17	112.04	5835.17	5.260	0.022	0.92
N67	24.39	411.01	17.15	384.99	0.793	0.373	1.00
N68	61.46	1895.28	48.74	1814.21	0.939	0.333	0.74
N69	44.89	1187.94	37.57	1116.08	0.264	0.607	0.89
N70	82.91	6027.17	112.04	5835.17	5.260	0.022	0.72

Supplementary Fig. 9 The two-sided chi-squared test is performed based on the frequency of two minor quartets between the observed gene trees and simulated gene trees (Phybase with ILS). The QD values are calculated by all quartet trees from each gene of 557 SCOGs. The theta value of each internal branch is marked near this branch in the left ASTRAL tree. The ancestral branch of Chlorophyta and Prasinodermophyta is labeled with green background throughout the row in the right table.

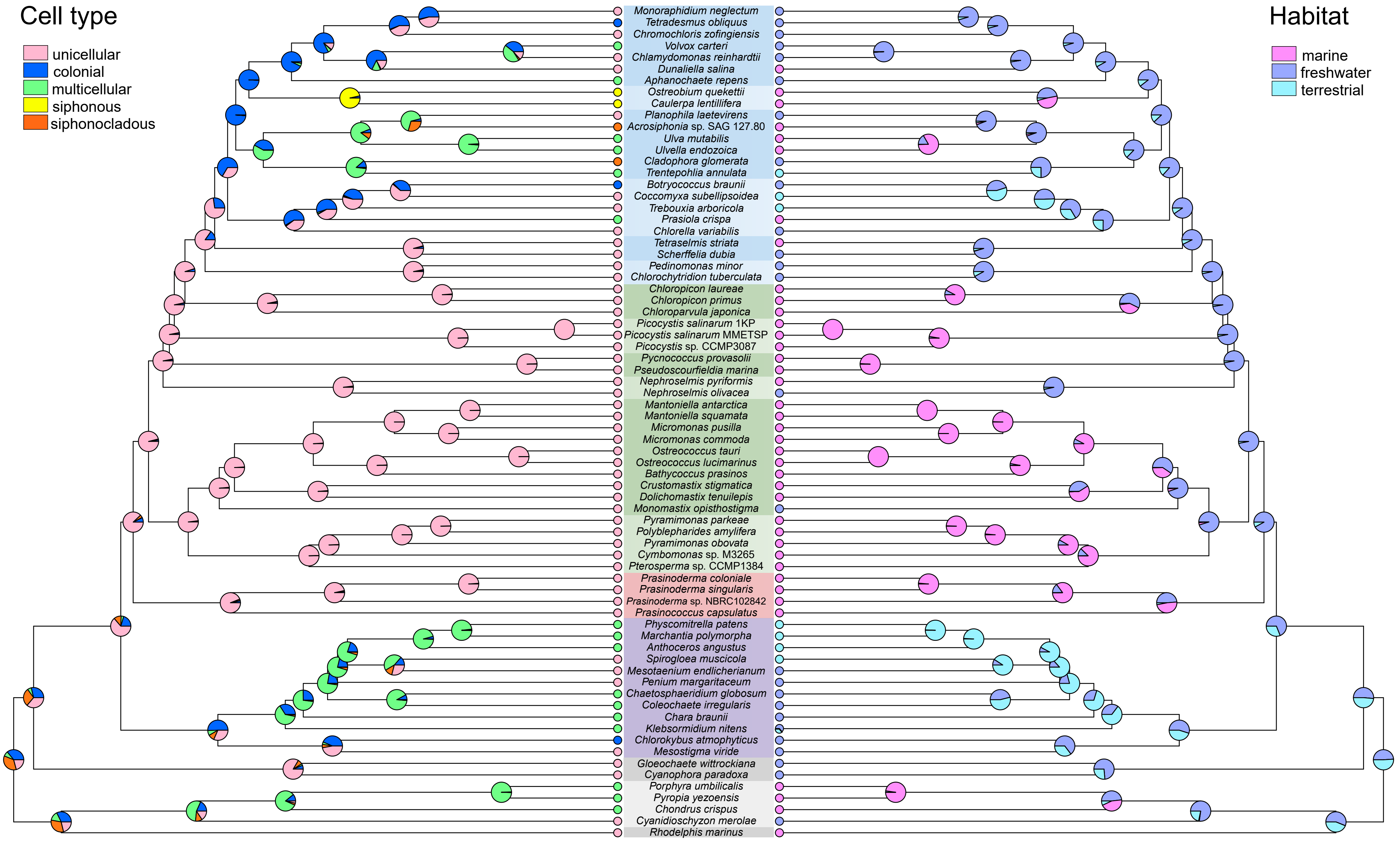

Supplementary Fig. 10 Ancestral state estimation of cell types (left) and habitat (right) by phytools are plotted on the ultrametric tree.

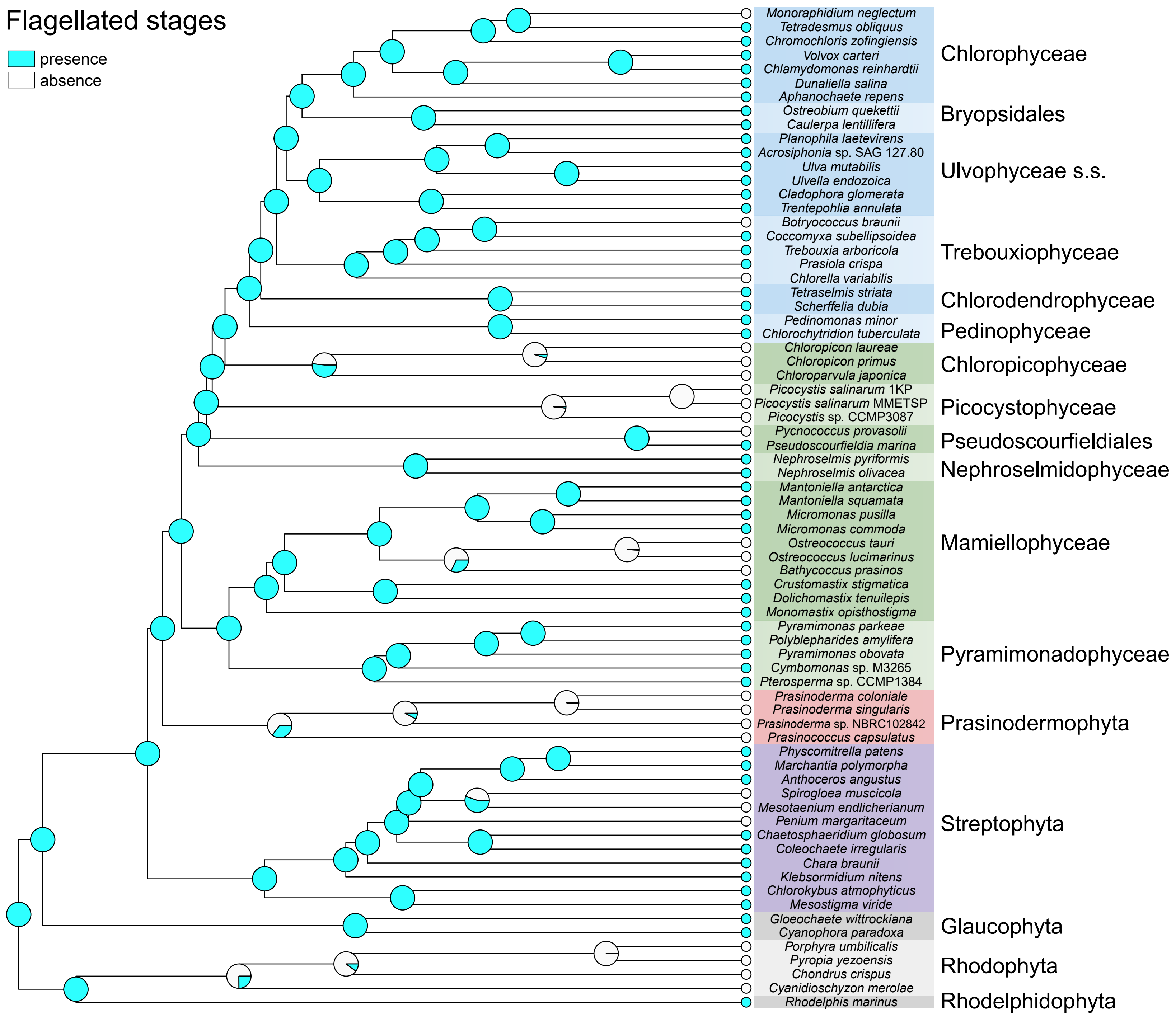

Supplementary Fig.11 Ancestral state estimation of the presence or absence of flagellate stages in the life cycle by phytools are plotted on the ultrametric tree.

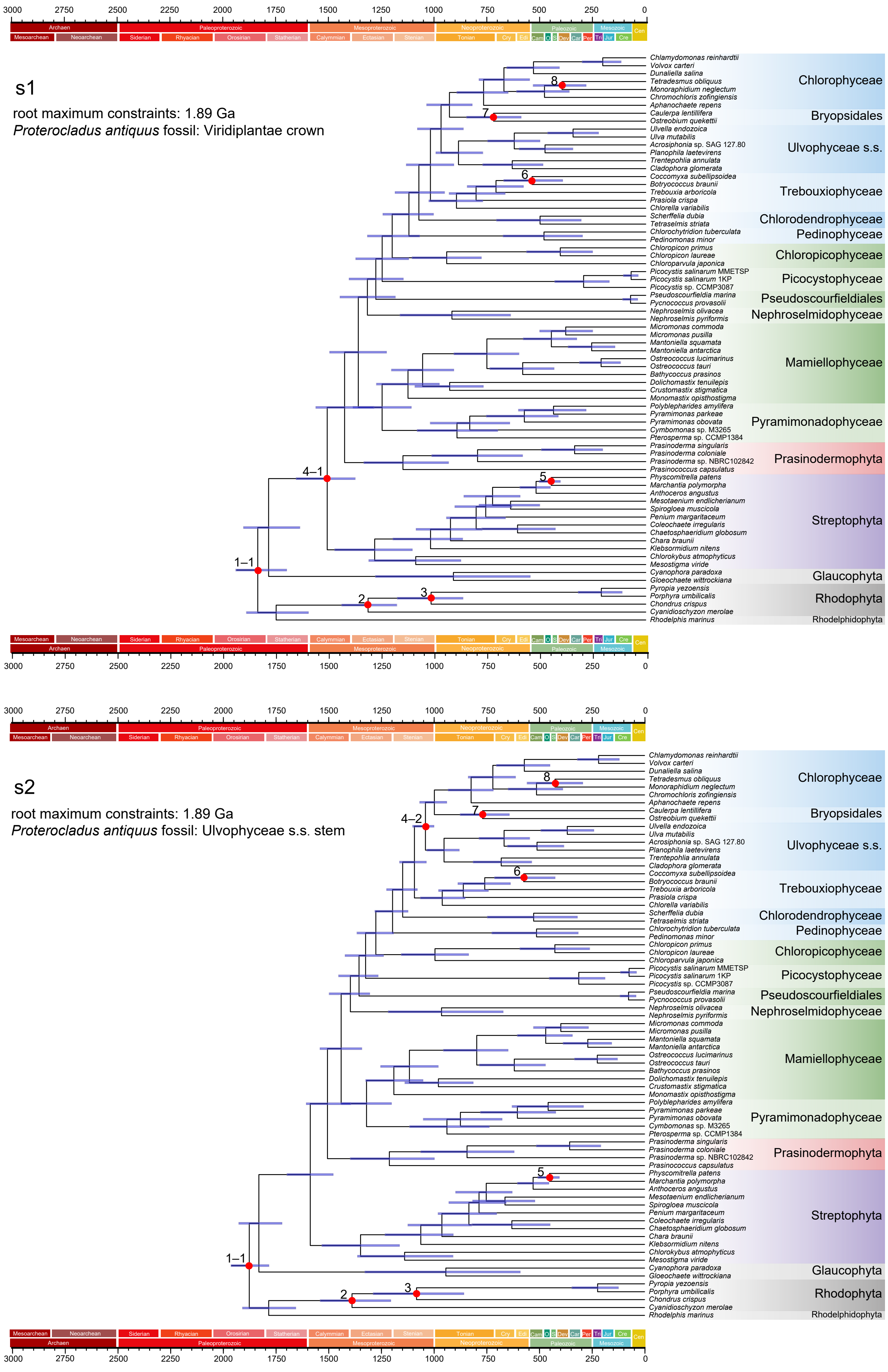

Supplementary Fig. 12 Time-calibrated tree based on the strategy 1 and 2. Each node represents the mean posterior time. The calibration nodes are represented by red dots, and their 95% credibility intervals are represented by the horizontal bars.

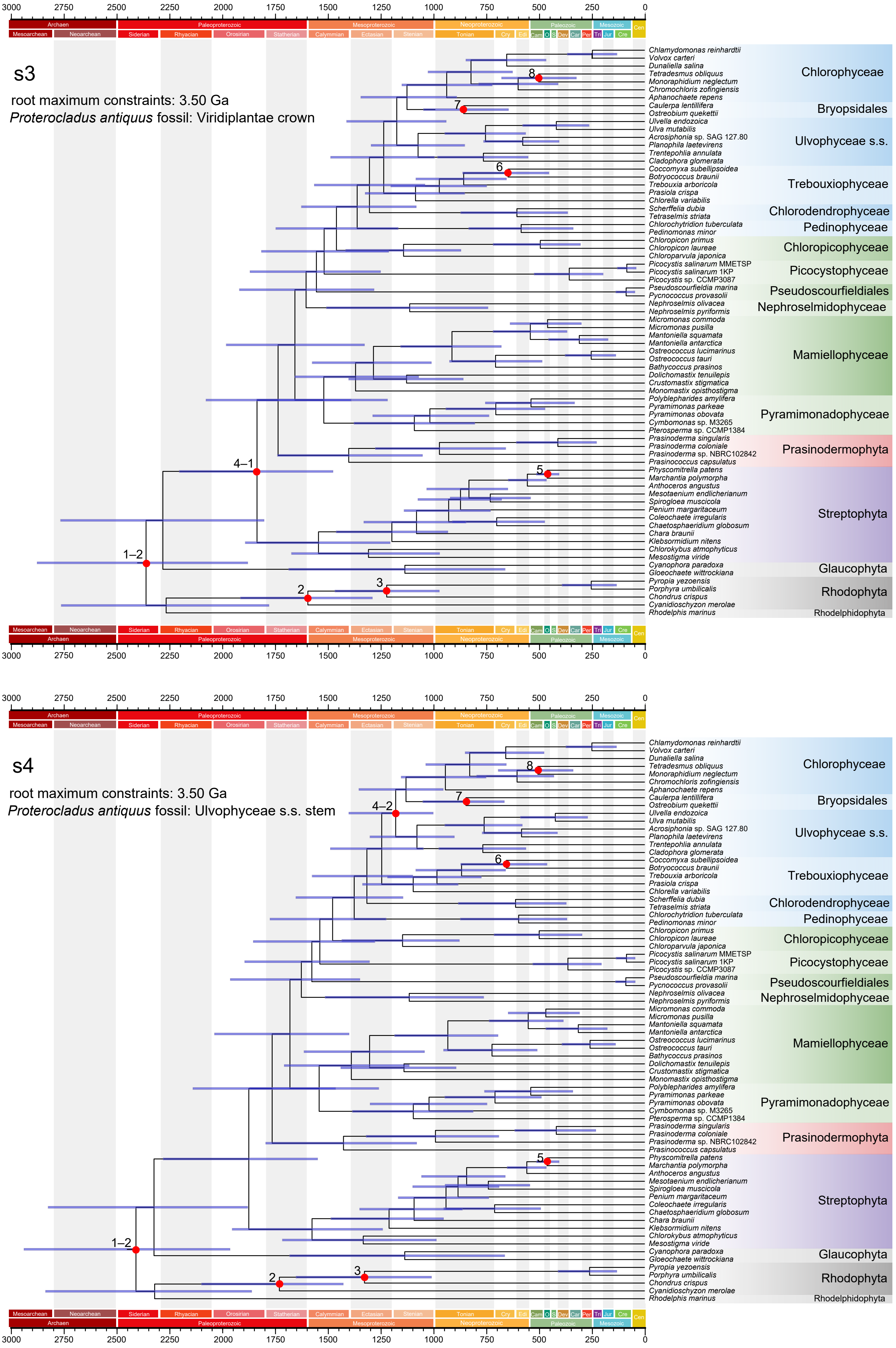

Supplementary Fig. 13 Time-calibrated tree based on the strategy 3 and 4. Each node represents the mean posterior time. The calibration nodes are represented by red dots, and their 95% credibility intervals are represented by the horizontal bars.

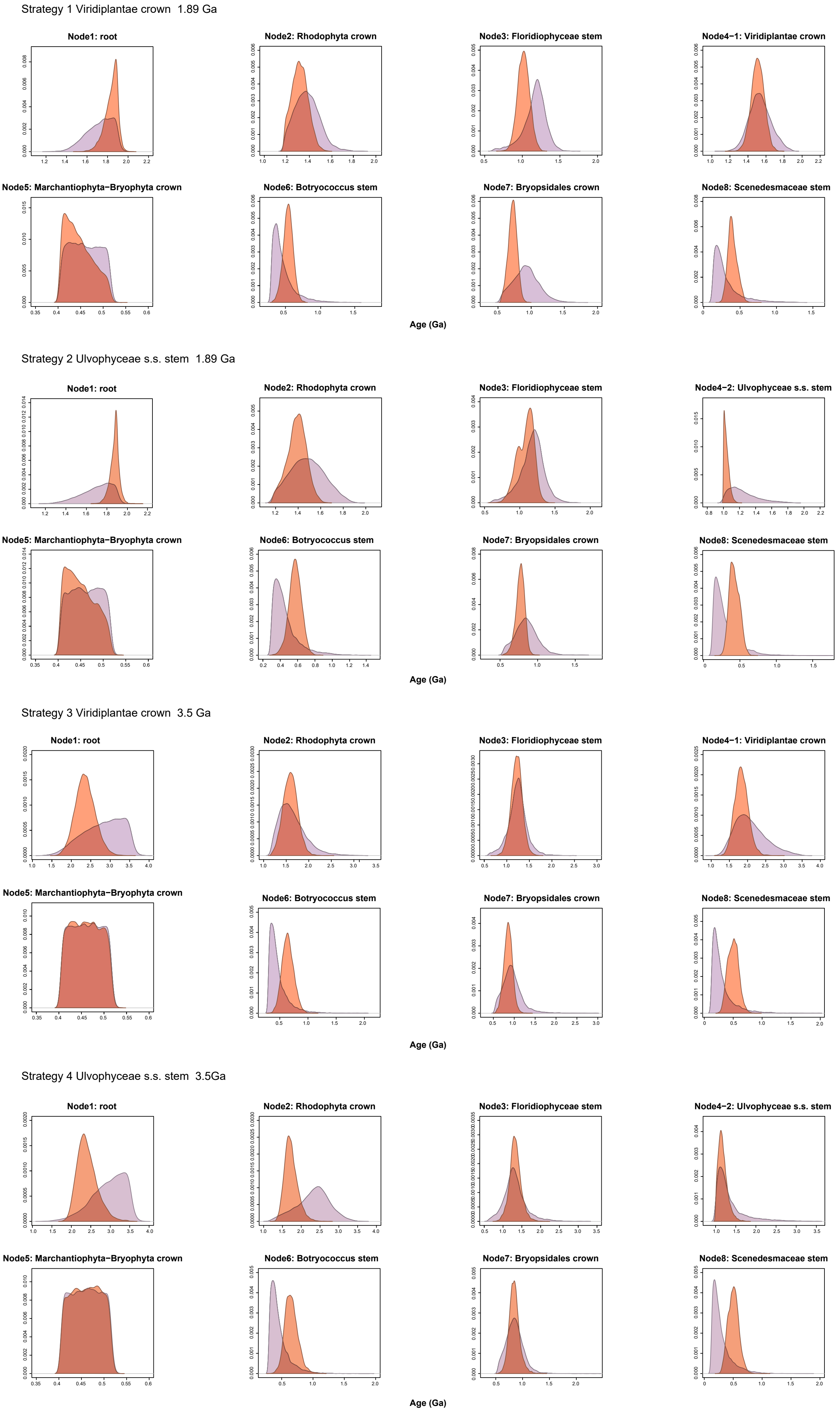

Supplementary Fig. 14 Comparison of the probability density distributions of the effective prior (purple) and posterior (orange) on calibration nodes. The horizontal axis represents time (Ga).

Supplementary Table 1 : Cyto-morphologies, habitats and the loss of flagellate stages of the 72 species (our taxon sampling of green plants) for ancestral state reconstruction.

	**Species**	**Cytomorphology of vegetative phase**	**Flagellate stages present in species**	**Habitat**
Pedinophyceae	*Pedinomonas minor*	unicellular[1]	yes[1]	freshwater[1]
	Chlorochytridion tuberculata	unicellular[2]	yes[2]	freshwater[3]
Chlorodendrophyceae	*Tetraselmis striata*	unicellular[4]	yes[5]	marine[6–8]
	Scherffelia dubia	unicellular[9]	yes[9]	freshwater[6,7]
Chlorophyceae	*Chromochloris zofingiensis*	unicellular[10]	yes[11]	freshwater[11]
	Monoraphidium neglectum	unicellular[7]	no[7]	freshwater[7]
	Tetradesmus obliquus	colonial[12]	yes[12]	freshwater[12]
	Chlamydomonas reinhardtii	unicellular[13]	yes[13]	freshwater[14]
	Dunaliella salina	unicellular[15]	yes[16]	marine(salt lakes)[15]
	Volvox carteri	multicellular[17]	yes[18]	freshwater[19]
	Aphanochaete repens	multicellular[20]	yes[7]	freshwater[7]
Trebouxiophyceae	*Chlorella variabilis*	unicellular[21]	no[21]	freshwater[22]
	Botryococcus braunii	colonial[23]	no[7]	freshwater[23]
	Trebouxia arboricola	unicellular[24]	yes[25]	terrestrial[24]
	Coccomyxa subellipsoidea	unicellular[26]	yes[26]	terrestrial[26]
	Prasiola crispa	multicellular[27]	yes[7]	marine (at or above high water mark) to fully terrestrial[28].
Ulvophyceae	*Caulerpa lentillifera*	siphonous[29]	yes[30]	marine[29]
	Ostreobium quekettii	siphonous[31]	yes[32]	marine[32]
	Ulva mutabilis	multicellular[33]	yes[33]	marine[7]
	Ulvella endozoica	multicellular[34]	yes[35]	marine[34]
	Cladophora glomerata	siphonocladous[36]	yes[36]	freshwater[36,37]
	Trentepohlia annulata	multicellular[38]	yes[38]	terrestrial[38]
	Acrosiphonia sp. SAG 127.80[1]	siphonocladous[39,40]	yes[7]	marine[40]
	Planophila laetevirens	unicellular[41]	yes [7,41]	freshwater and soil[41]
Prasinodermophyta	*Prasinococcus capsulatus*	unicellular[42]	no[42]	marine[42]
	Prasinoderma coloniale	unicellular[43]	no[43]	marine[43]
	Prasinoderma singularis	unicellular[44]	no[44]	marine[44]
	Prasinoderma sp. NBRC102842	unicellular[45]	no[45]	marine[45]
Mamiellophyceae	*Bathycoccus prasinos*	unicellular[46,47]	no[46,47]	marine[47]
	Ostreococcus lucimarinus	unicellular[47]	no[47]	marine[47]
	Ostreococcus tauri	unicellular[48]	no[48]	marine[47]
	Mantoniella antarctica	unicellular[49]	yes[49]	marine[47]
	Mantoniella squamata	unicellular[50]	yes[50]	marine[47]
	Micromonas commoda	unicellular[51]	yes[51]	marine[47]

[1] this type of multinucleate cells probably evolved independently from the Cladophorales

	Micromonas pusilla	unicellular[51]	yes[51]	marine[47]
	Monomastix opisthostigma	unicellular[47]	yes[47]	freshwater[7,47]
	Crustomastix stigmatica	unicellular[52]	yes[52]	marine[47,52]
	Dolichomastix tenuilepis	unicellular[53]	yes[53]	marine[47,53]
Pseudoscourfieldiales	*Pseudoscourfieldia marina*	unicellular[54,55]	yes[54,55]	marine[55]
	Pycnococcus provasolii	unicellular[56]	no(rarely)[56]	marine[55,56]
Pyramimonadophyceae	*Pterosperma* sp. CCMP1384	unicellular[57]	yes[57]	marine[57]
	Cymbomonas sp. M3265	unicellular[58]	yes[58]	marine[58]
	Pyramimonas obovata	unicellular[59]	yes[59]	marine[60]
	Pyramimonas parkeae	unicellular[61]	yes[61]	marine[60,61]
	Polyblepharides amylifera	unicellular[62,63]	yes[62,63]	marine[63]
Nephroselmidophyceae	*Nephroselmis olivacea*	unicellular[55]	yes[55]	freshwater[55]
	Nephroselmis pyriformis	unicellular[55]	yes[55]	marine[55]
Chloropicophyceae	*Chloropicon laureae*	unicellular[64]	no[64]	marine[64]
	Chloropicon primus	unicellular[64]	no[64]	marine[64]
	Chloroparvula japonica	unicellular[64]	no[64]	marine[64]
Picocystophyceae	*Picocystis salinarum* 1KP	unicellular[65]	no (unkonown)[65]	marine[65]
	Picocystis salinarum MMETSP	unicellular[65]	no (unkonown)[65]	marine[65]
	Picocystis sp. CCMP3087	unicellular[65]	no (unkonown)[65]	marine[65]
Charophyta	*Chlorokybus atmophyticus*	colonial[66,67]	yes[68,69]	moist terrestrial/freshwater[68]
	Mesostigma viride	unicellular[70]	yes[69,70]	freshwater[70]
	Klebsormidium nitens	multicellular[70,71]	yes[69,72]	terrestrial and freshwater[73]
	Chara braunii	multicellular[74]	yes[69,74]	freshwater[75]
	Coleochaete irregularis	multicellular[76]	yes[69,77]	freshwater[76]
	Chaetosphaeridium globosum	multicellular[78]	yes[69,79]	freshwater[7]
	Penium margaritaceum	unicellular[80]	no[69,81]	freshwater[81]
	Mesotaenium endlicherianum	unicellular[82,83]	no[69,81]	freshwater[81]
	Spirogloea muscicola	unicellular[82]	no[69,81]	terrestrial[82]
Anthocerotophyta	*Anthoceros angustus*	multicellular[84]	yes[84]	terrestrial[85]
Bryophyta	*Physcomitrella patens*	multicellular[86]	yes[87]	terrestrial[85]
Marchantiophyta	*Marchantia polymorpha*	multicellular[88]	yes[88]	terrestrial[85]
Glaucophyta	*Cyanophora paradoxa*	unicellular[89]	yes[89]	freshwater[89]
	Gloeochaete wittrockiana	unicellular[90]	yes[91]	freshwater[92]
Rhodophyta	*Chondrus crispus*	multicellular[93]	no[94]	marine[93]
	Porphyra umbilicalis	multicellular[95]	no[94]	marine[7]
	Pyropia yezoensis	multicellular[96]	no[94]	marine[7,96]
	Cyanidioschyzon merolae	unicellular[97]	no[94]	freshwater[98]
Rhodelphidophyta	*Rhodelphis marinus*	unicellular[99]	yes[99]	marine[99]

Supplementary Table 2: Comparison of posterior divergence times of major green plants using different strategies.

clade	Estimated age (Ga) (95% CI)			
	s1	s2	s3	s4
Root	1.94–1.70	1.96–1.78	2.88–1.88	2.94–1.96
Viridiplantae	1.65–1.38	1.70–1.48	2.20–1.48	2.28–1.55
Prasinodermophyta	1.33–0.93	1.40–1.00	1.74–1.05	1.79–1.08
Chlorophyta	1.50–1.23	1.54–1.34	1.98–1.33	2.04–1.40
prasinophytes	1.39–1.11	1.44–1.20	1.83–1.22	1.26–1.28
core Chlorophyta	1.24–1.01	1.28–1.12	1.62–1.08	1.65–1.14
Ulvophyceae s.s.	0.99–0.77	1.03–0.88	1.29–0.85	1.30–0.90

Reference

1. Xu, Y. *et al.* Chromosome-level genome of *Pedinomonas minor* (Chlorophyta) unveils adaptations to abiotic stress in a rapidly fluctuating environment. *New Phytol.* **235**, 1409–1425 (2022).
2. Ricketts, T. R. The pigments of the phytoflagellates, *Pedinomonas minor* and *Pedinomonas tuberculata*. *Phytochemistry* **6**, 19–24 (1967).
3. Ettl, H. & Gärtner, G. *Syllabus der Boden-, Luft- und Flechtenalgen*. (Springer Berlin Heidelberg, 2014). doi:10.1007/978-3-642-39462-1.
4. Hori, T., Norris, R. E. & Chihara, M. Studies on the ultrastructure and taxonomy of the genusTetraselmis (Prasinophyceae): I. SubgenusTetraselmis. *Bot. Mag. Tokyo* **95**, 49–61 (1982).
5. Gödel, S., Becker, B. & Melkonian, M. Flagellar membrane proteins of *Tetraselmis striata* Butcher (Chlorophyta). *Protist* **151**, 147–159 (2000).
6. Leliaert, F. *et al.* Phylogeny and molecular evolution of the green algae. *Crit. Rev. Plant Sci.* **31**, 1–46 (2012).
7. Guiry, M. D & Guiry, G. M. AlgaeBase. World-wide electronic publication, National University of Ireland, Galway. https://www.algaebase.org (Accessed 22 September 2022).
8. Pagarete, A., Grébert, T., Stepanova, O., Sandaa, R.-A. & Bratbak, G. Tsv-N1: A Novel DNA Algal Virus that Infects Tetraselmis striata. *Viruses* **7**, 3937–3953 (2015).
9. Wustman, B. A., Melkonian, M. & Becker, B. A study of cell wall and flagella formation during cell division in the scaly green alga, *Scherffelia dubia* (Chlorophyta). *J. Phycol.* **40**, 895–910 (2004).
10. Roth, M. S. *et al.* Chromosome-level genome assembly and transcriptome of the green alga *Chromochloris zofingiensis* illuminates astaxanthin production. *Proc. Natl. Acad. Sci.* **114**, E4296–E4305 (2017).
11. Fučíková, K. & Lewis, L. A. Intersection of *Chlorella*, *Muriella* and *Bracteacoccus*: Resurrecting the genus *Chromochloris* Kol et Chodat (Chlorophyceae, Chlorophyta). *Fottea* **12**, 83–93 (2012).
12. Cain, J. R. & Trainor, F. B. Regulation of gametogenesis in *Scenedesmus obliquus* (Chlorophyceae). *J. Phycol.* **12**, (1976).
13. Rochaix, J.-D. *Chlamydomonas reinhardtii*. in *Brenner's Encyclopedia of Genetics (Second Edition)* (eds. Maloy, S. & Hughes, K.) 521–524 (Academic Press, 2013). doi:https://doi.org/10.1016/B978-0-12-374984-0.00230-8.
14. Sasso, S., Stibor, H., Mittag, M. & Grossman, A. R. From molecular manipulation of domesticated *Chlamydomonas reinhardtii* to survival in nature. *eLife* **7**, e39233 (2018).
15. Zhao, R. *et al.* Analysis of expressed sequence tags from the green alga *Dunaliella salina*(chlorophyta). *J. Phycol.* **47**, 1454–1460 (2011).
16. Jia, Y., Xue, L., Li, J. & Liu, H. Isolation and proteomic analysis of the halotolerant alga *Dunaliella salina* flagella using shotgun strategy. *Mol. Biol. Rep.* **37**, 711–716 (2010).

17. Herron, M. D. Origins of multicellular complexity: *Volvox* and the volvocine algae. *Mol. Ecol.* **25**, 1213–1223 (2016).
18. Kochert, G. & Olson, L. W. Ultrastructure of *Volvox carteri*. *Arch. Für Mikrobiol.* **74**, 19–30 (1970).
19. Halder, N. & Sinha, S. N. *Volvox carteri* F. Stein- a new report from West Bengal, India. in (2016).
20. Keshri, J. & Sarma, P. The genus *Aphanochaete A. Braun* (Chaetophorales: Chlorophyta) in West Bengal, India. *Geophytology,* **33**, 35–37 (2004).
21. Blanc, G. *et al.* The *Chlorella variabilis* NC64A genome reveals adaptation to photosymbiosis, coevolution with viruses, and cryptic sex. *Plant Cell* **22**, 2943–2955 (2010).
22. Fott, B. A monograph of the genus *Chlorella*. The fresh water species. *Stud. Phycol.* 10–70 (1969).
23. Metzger, P. & Largeau, C. *Botryococcus braunii* : a rich source for hydrocarbons and related ether lipids. *Appl. Microbiol. Biotechnol.* **66**, 486–496 (2005).
24. Muggia, L. *et al.* Formally described species woefully underrepresent phylogenetic diversity in the common lichen photobiont genus *Trebouxia* (Trebouxiophyceae, Chlorophyta): An impetus for developing an integrated taxonomy. *Mol. Phylogenet. Evol.* **149**, 106821 (2020).
25. Friedl, T. Comparative ultrastructure of pyrenoids in *Trebouxia* (Microthamniales, Chlorophyta). *Plant Syst. Evol.* **164**, 145–159 (1989).
26. Acton, E. *Coccomyxa subellipsoidea*, a new member of the Palmellaceae. *Ann. Bot.* **os-23**, 573–578 (1909).
27. Richter, D., Matuła, J., Urbaniak, J., Waleron, M. & Czerwik-Marcinkowska, J. Molecular, morphological and ultrastructural characteristics of *Prasiola crispa* (Lightfoot) Kützing (Chlorophyta) from Spitsbergen (Arctic). *Polar Biol.* **40**, 379–397 (2017).
28. Brodie, J. A., Maggs, C. & John, D. M. *Green seaweeds of Britain and Ireland.* (British Phycological Society, 2007).
29. Arimoto, A. *et al.* A siphonous macroalgal genome suggests convergent functions of homeobox genes in algae and land plants. *DNA Res.* **26**, 183–192 (2019).
30. Liu, T. *Atlas of common macroalgae from the South China Sea*. (China Ocean Press, 2018).
31. Tandon, K. *et al.* Every refuge has its price: *Ostreobium* as a model for understanding how algae can live in rock and stay in business. *Semin. Cell Dev. Biol.* S1084952122000775 (2022) doi:10.1016/j.semcdb.2022.03.010.
32. Kornmann, P. & Sahling, P.-H. *Ostreobium quekettii* (Codiales, Chlorophyta). *Helgoländer Meeresunters.* **34**, 115–122 (1980).
33. BråTen, T. The ultrastructure of fertilization and zygote formation in the green alga *Ulva mutabilis* Føyn. *J. Cell Sci.* **9**, 621–635 (1971).

34. Soares, L. P. *et al.* New insights on the distribution and habitat of *Ulvella endozoica* (Ulvellaceae, Chlorophyta) in the tropical Southwestern Atlantic, based on thallus ontogeny in culture and DNA barcoding. *Mar. Biodivers.* **51**, 12 (2021).
35. Goldberg, W. M., Makemson, J. C. & Colley, S. B. *Entocladia endozoica* sp. nov., a pathogenic chlorophyte: structure, life history, physiology, and effect on its coral host. *Biol. Bull.* **166**, 368–383 (1984).
36. Whitton, B. A. Biology of *Cladophora* in freshwaters. *Water Res.* **4**, 457–476 (1970).
37. John, D. M. Filamentous and plantlike green algae. in *Freshwater Algae of North America* (eds. Wehr, J. D. & Sheath, R. G.) 311–352 (Academic Press, 2003).
38. Rindi, F. & Guiry, M. D. Diversity, life History, and ecology of *Trentepohlia* and *Printzina* (Trentepohliales, Chlorophyta) in urban habitats in western Ireland. *J. Phycol.* **38**, 39–54 (2002).
39. Lokhorst, G. M. & Star, W. Fine structure of mitosis and cytokinesis in *Urospora* (Acrosiphoniales, Chlorophyta). *Protoplasma* **117**, 142–153 (1983).
40. Rorrer, G. L. & Cheney, D. P. Bioprocess engineering of cell and tissue cultures for marine seaweeds. *Aquac. Eng.* **32**, 11–41 (2004).
41. Friedl, T. & O'Kelly, C. J. Phylogenetic relationships of green algae assigned to the genus *Planophila* (Chlorophyta): evidence from 18S rDNA sequence data and ultrastructure. *Eur. J. Phycol.* **37**, 373–384 (2002).
42. Miyashita, H., Ikemoto, H., Kurano, N., Miyachi, S. & Chihara, M. *Prasinococcus capsulatus* gen. et sp. nov., a new marine coccoid prasinophyte. *J. Gen. Appl. Microbiol.* **39**, 571–582 (1993).
43. Hasegawa, T. *et al. Prasinoderma coloniale* gen. et sp. nov., a new pelagic coccoid prasinophyte from the western Pacific Ocean. *Phycologia* **35**, 170–176 (1996).
44. Jouenne, F. *et al. Prasinoderma singularis* sp. nov. (Prasinophyceae, Chlorophyta), a solitary coccoid prasinophyte from the South-East Pacific Ocean. *Protist* **162**, 70–84 (2011).
45. Leliaert, F. *et al.* Chloroplast phylogenomic analyses reveal the deepest-branching lineage of the Chlorophyta, Palmophyllophyceae class. nov. *Sci. Rep.* **6**, 25367 (2016).
46. Eikrem, W. & Throndsen, J. The ultrastructure of *Bathycoccus* gen. nov. and *B. prasinos* sp. nov., a non-motile picoplanktonic alga (Chlorophyta, Prasinophyceae) from the Mediterranean and Atlantic. *Phycologia* **29**, 344–350 (1990).
47. Marin, B. & Melkonian, M. Molecular phylogeny and classification of the Mamiellophyceae class. nov. (Chlorophyta) based on sequence comparisons of the nuclear- and plastid-encoded rRNA operons. *Protist* **161**, 304–336 (2010).
48. Chrétiennot-Dinet, M.-J. *et al.* A new marine picoeucaryote: *Ostreococcus tauri* gen. et sp. nov. (Chlorophyta, Prasinophyceae). *Phycologia* **34**, 285–292 (1995).
49. Marchant, H. J., Buck, K. R., Garrison, D. L. & Thomsen, H. A. *Mantoniella* in antarctic waters including the description of *M. antarctica* sp. nov. (prasinophyceae). *J. Phycol.* **25**, 167–174 (1989).

50. Barlow, S. B. & Cattolico, R. A. Fine structure of the scale-covered green flagellate *Mantoniella squamata* (Manton et Parke) Desikachary. *Br. Phycol. J.* **15**, 321–333 (1980).
51. van Baren, M. J. *et al.* Evidence-based green algal genomics reveals marine diversity and ancestral characteristics of land plants. *BMC Genomics* **17**, 267 (2016).
52. Zingone, A. *et al.* Phylogenetic position of *Crustomastix stigmatica* sp. nov. and *Dolichomastix tenuilepis* in relation to the Mamiellales (Prasinophyceae, Chlorophyta). *J. Phycol.* **38**, 1024–1039 (2002).
53. Throndsen, J. & Zingone, A. *Dolichomastix tenuilepis* sp. nov., a first insight into the microanatomy of the genus *Dolichomastix* (Mamiellales, Prasinophyceae, Chlorophyta). *Phycologia* **36**, 244–254 (1997).
54. Irene Manton. Observations on the microanatomy of *Scourfieldia marina* Throndsen and *Scourfieldia caeca* (Korsch.) Belcher et Swale. *Arch. Für Protistenkd.* **117**, 358–368 (1975).
55. Nakayama, T., Suda, S., Kawachi, M. & Inouye, I. Phylogeny and ultrastructure of *Nephroselmis* and *Pseudoscourfieldia* (Chlorophyta), including the description of *Nephroselmis anterostigmatica* sp. nov. and a proposal for the Nephroselmidales ord. nov. *Phycologia* **46**, 680–697 (2007).
56. Guillard, R. R. L., Keller, M. D., O'Kelly, C. J. & Floyd, G. L. *Pycnococcus provasolii* gen. et sp. nov., a coccoid prasinoxanthin-containing phytoplankter from the Western North Atlantic and Gulf of Mexico. *J. Phycol.* **27**, 39–47 (1991).
57. Parke, M., Boalch, G. T., Jowett, R. & Harbour, D. S. The genus *Pterosperma* (Prasinophyceae): species with a single equatorial ala. *J. Mar. Biol. Assoc. U. K.* **58**, 239–276 (1978).
58. Moestrup, Ø., Inouye, I. & Hori, T. Ultrastructural studies on *Cymbomonas tetramitiformis* (Prasinophyceae). I. General structure, scale microstructure, and ontogeny. *Can. J. Bot.* **81**, 657–671 (2003).
59. Melkonian, M. The flagellar apparatus of the scaly green flagellate *Pyramimonas obovata*: Absolute configuration. *Protoplasma* **108**, 341–355 (1981).
60. Harðardóttir, S., Lundholm, N., Moestrup, Ø. & Nielsen, T. G. Description of *Pyramimonas diskoicola* sp. nov. and the importance of the flagellate *Pyramimonas* (Prasinophyceae) in Greenland sea ice during the winter–spring transition. *Polar Biol.* **37**, 1479–1494 (2014).
61. Norris, R. E. & Pearson, B. R. Fine structure of *Pyramimonas parkeae*, sp. nov. (Chlorophyta, Prasinophyceae). *Arch. Für Protistenkd.* (1975).
62. Gardiner, W. E. & Hargraves, P. E. Life cycle, seasonality, and effects of temperature and salinity on growth and survival of *Pyramimonas amylifera* (Prasinophyceae). *J Phycol U. S.* **14**, (1978).
63. Woods, J. K. & Triemer, R. E. Mitosis in the octoflagellate prasinophyte, *Pyramimonas amylifera* (Chlorophyta). *J. Phycol.* **17**, 81–90 (1981).
64. Lopes dos Santos, A. *et al.* Chloropicophyceae, a new class of picophytoplanktonic prasinophytes. *Sci. Rep.* **7**, 14019 (2017).

65. Lewin, R. A., Krienitz, L., Goericke, R., Takeda, H. & Hepperle, D. *Picocystis salinarum* gen. et sp. nov. (Chlorophyta) – a new picoplanktonic green alga. *Phycologia* **39**, 560–565 (2000).
66. Škaloud, P. Species composition and diversity of aero-terrestrial algae and cyanobacteria of the Boreč Hill ventaroles. *Fottea* **9**, 65–80 (2009).
67. Wang, S. *et al.* Genomes of early-diverging streptophyte algae shed light on plant terrestrialization. *Nat. Plants* **6**, 95–106 (2020).
68. Lokhorst, G. M., Sluiman, H. J. & Star, W. The ultrastructure of mitosis and cytokinesis in the sarcinoid *Chlorokybus atmophyticus* (Chlorophyta, Charophyceae) revealed by rapid freeze fixation and freeze substitution. *J. Phycol.* **24**, 237–248 (1988).
69. Lewis, L. A. & McCourt, R. M. Green algae and the origin of land plants. *Am. J. Bot.* **91**, 1535–1556 (2004).
70. Liang, Z. *et al. Mesostigma viride* genome and transcriptome provide insights into the origin and evolution of Streptophyta. *Adv. Sci.* **7**, 1901850 (2020).
71. Hori, K. *et al. Klebsormidium flaccidum* genome reveals primary factors for plant terrestrial adaptation. *Nat. Commun.* **5**, 3978 (2014).
72. Rindi, F., Ryšánek, D. & Škaloud, P. Problems of epitypification in morphologically simple green microalgae: a case study of two widespread species of *Klebsormidium* (Klebsormidiophyceae, Streptophyta). *Fottea* **17**, 78–88 (2017).
73. Škaloud, P. Variation and taxonomic significance of some morphological features in European strains of *Klebsormidium* (Klebsormidiophyceae, Streptophyta). *Nova Hedwig.* **83**, 533–550 (2006).
74. Nishiyama, T. *et al.* The *Chara* Genome: sKnecondary complexity and implications for plant terrestrialization. *Cell* **174**, 448-464.e24 (2018).
75. Urbaniak, J. Distribution of *Chara braunii* Gmellin 1826 (Charophyta) in Poland. *Acta Soc. Bot. Pol.* **76**, 313–320 (2011).
76. Graham, L. E. The occurrence, evolution, and phylogenetic significance of parenchyma in *Coleochaete* Bréb. (Chlorophyta). *Am. J. Bot.* **69**, 447–454 (1982).
77. Haig, D. *Coleochaete* and the origin of sporophytes. *Am. J. Bot.* **102**, 417–422 (2015).
78. Thompson, R. H. Sexual reproduction in *Chaetosphaeridium globosum* (Nordst.) Klebahn (Chlorophyceae) and description of a species new to science. *J. Phycol.* **5**, 285–290 (1969).
79. Moestrup, Ø. Ultrastructure of the scale-covered zoospores of the green alga *Chaetosphaeridium*, a possible ancestor of the higher plants and bryophytes. *Biol. J. Linn. Soc.* **6**, 111–125 (1974).
80. Raimundo, S. C. *et al.* Isolation and manipulation of protoplasts from the unicellular green alga *Penium margaritaceum*. *Plant Methods* **14**, 18 (2018).
81. Guiry, M. Taxonomy and nomenclature of the Conjugatophyceae (= Zygnematophyceae). *ALGAE* **28**, (2013).
82. Cheng, S. *et al.* Genomes of subaerial Zygnematophyceae provide insights into land plant evolution. *Cell* **179**, 1057-1067.e14 (2019).

83. Del Cortona, A. *et al.* Neoproterozoic origin and multiple transitions to macroscopic growth in green seaweeds. *Proc. Natl. Acad. Sci.* **117**, 2551–2559 (2020).
84. Frangedakis, E. *et al.* The hornworts: morphology, evolution and development. *New Phytol.* **229**, 735–754 (2021).
85. Longton, R. E. Preface: Bryophytes in terrestrial ecosystems. *Bot. J. Linn. Soc.* **104**, 1–1 (1990).
86. Falz, A.-L. & Müller-Schüssele, S. J. *Physcomitrella* as a model system for plant cell biology and organelle–organelle communication. *Curr. Opin. Plant Biol.* **52**, 7–13 (2019).
87. Horst, N. A. & Reski, R. Microscopy of *Physcomitrella patens* sperm cells. *Plant Methods* **13**, 33 (2017).
88. Shimamura, M. *Marchantia polymorpha* : Taxonomy, Phylogeny and Morphology of a Model System. *Plant Cell Physiol.* **57**, 230–256 (2016).
89. Price, D. C. *et al. Cyanophora paradoxa* genome elucidates origin of photosynthesis in algae and plants. *Science* **335**, 843–847 (2012).
90. Price, D. C., Steiner, J. M., Yoon, H. S., Bhattacharya, D. & Löffelhardt, W. Glaucophyta. in *Handbook of the Protists* (eds. Archibald, J. M. et al.) 1–65 (Springer International Publishing, 2016). doi:10.1007/978-3-319-32669-6_42-1.
91. Kies, L. Untersuchungen zur Feinstruktur und taxonomischen Einordnung vonGloeochaete wittrockiana, einer apoplastidalen capsalen Alge mit blaugrünen Endosymbionten (Cyanellen). *Protoplasma* **87**, 419–446 (1976).
92. Jackson, C. J. & Reyes-Prieto, A. The mitochondrial genomes of the glaucophytes *Gloeochaete wittrockiana* and *Cyanoptyche gloeocystis*: Multilocus phylogenetics suggests a monophyletic Archaeplastida. *Genome Biol. Evol.* **6**, 2774–2785 (2014).
93. Collén, J. *et al. Chondrus crispus* – a present and historical model organism for red seaweeds. in *Advances in Botanical Research* vol. 71 53–89 (Elsevier, 2014).
94. Hoek, C. V. D., Mann, D. G. V. D. & Jahns, H. M. Algae. An introduction to phycology. *J. North Am. Benthol. Soc.* **16**, (1995).
95. Gantt, E. *et al. Porphyra* : complex life histories in a harsh environment: *P. umbilicalis*, an intertidal red alga for genomic analysis. in *Red Algae in the Genomic Age* (eds. Seckbach, J. & Chapman, D. J.) vol. 13 129–148 (Springer Netherlands, 2010).
96. Mikami, K., Li, C., Irie, R. & Hama, Y. A unique life cycle transition in the red seaweed *Pyropia yezoensis* depends on apospory. *Commun. Biol.* **2**, 299 (2019).
97. Miyagishima, S.-Y. & Tanaka, K. The unicellular red alga *Cyanidioschyzon merolae*— the simplest model of a photosynthetic eukaryote. *Plant Cell Physiol.* **62**, 926–941 (2021).
98. Hirooka, S. *et al.* Efficient open cultivation of cyanidialean red algae in acidified seawater. *Sci. Rep.* **10**, 13794 (2020).
99. Gawryluk, R. M. R. *et al.* Non-photosynthetic predators are sister to red algae. *Nature* **572**, 240–243 (2019).
