# Supplementary material for: Phylotranscriptomics unveil a Paleoproterozoic-Mesoproterozoic origin and deep relationships of the Viridiplantae
Source: Nat Commun. 2023 Sep 11;14:5542. doi: 10.1038/s41467-023-41137-5 (PMC10495350; doi:10.1038/s41467-023-41137-5)
Supplement: Supplementary file 3 — Description of Additional Supplementary Files [file 41467_2023_41137_MOESM3_ESM.pdf]

Description of Additional Supplementary Files

File Name: Supplementary Data 1
Description: Taxon sampling of the nuclear and plastid datasets were used in this study.
