# Supplementary material for: Phylotranscriptomics unveil a Paleoproterozoic-Mesoproterozoic origin and deep relationships of the Viridiplantae
Source: Nat Commun. 2023 Sep 11;14:5542. doi: 10.1038/s41467-023-41137-5 (PMC10495350; doi:10.1038/s41467-023-41137-5)
Supplement: Supplementary file 5 — Reporting Summary [file 41467_2023_41137_MOESM5_ESM.pdf]

nature portfolio

Corresponding author(s): Bojian Zhong
Last updated by author(s): Aug 10, 2023

Reporting Summary

Nature Portfolio wishes to improve the reproducibility of the work that we publish. This form provides structure for consistency and transparency in reporting. For further information on Nature Portfolio policies, see our Editorial Policies and the Editorial Policy Checklist.

Statistics

For all statistical analyses, confirm that the following items are present in the figure legend, table legend, main text, or Methods section.

n/a	Confirmed	
☐	☒	The exact sample size (*n*) for each experimental group/condition, given as a discrete number and unit of measurement
☒	☐	A statement on whether measurements were taken from distinct samples or whether the same sample was measured repeatedly
☐	☒	The statistical test(s) used AND whether they are one- or two-sided<br>*Only common tests should be described solely by name; describe more complex techniques in the Methods section.*
☒	☐	A description of all covariates tested
☒	☐	A description of any assumptions or corrections, such as tests of normality and adjustment for multiple comparisons
☐	☒	A full description of the statistical parameters including central tendency (e.g. means) or other basic estimates (e.g. regression coefficient) AND variation (e.g. standard deviation) or associated estimates of uncertainty (e.g. confidence intervals)
☐	☒	For null hypothesis testing, the test statistic (e.g. *F*, *t*, *r*) with confidence intervals, effect sizes, degrees of freedom and *P* value noted<br>*Give P values as exact values whenever suitable.*
☐	☒	For Bayesian analysis, information on the choice of priors and Markov chain Monte Carlo settings
☒	☐	For hierarchical and complex designs, identification of the appropriate level for tests and full reporting of outcomes
☒	☐	Estimates of effect sizes (e.g. Cohen's *d*, Pearson's *r*), indicating how they were calculated

Our web collection on statistics for biologists contains articles on many of the points above.

Software and code

Policy information about availability of computer code

Data collection	No software was used in data collection.
Data analysis	Softwares used for data analysis include: OrthoFinder v2.4.1, HMMER v3.3.1, CD-HIT v4.8.1, MAFFT v7.471, trimAl v1.4, TreeShrink v1.3.2, RAxML v8.2.11, ASTRAL v5.7.4, Newick Utilities v1.6, IQ-TREE v2.0.7, Phyparts, PhyPartsPieCharts, ETE3, AstralPlane v0.1.1, TreSpEx v1.1, RAxML-NG v.1.2.0, TREESPACE v1.1.4.2, Phybase v.1.5, Quartet Sampling v1.3.1, phytools R package, SortaDate, PAML package v4.9j, Tracer v. 1.7.2, MCMCTreeR, phytools v1.5.1.

For manuscripts utilizing custom algorithms or software that are central to the research but not yet described in published literature, software must be made available to editors and reviewers. We strongly encourage code deposition in a community repository (e.g. GitHub). See the Nature Portfolio guidelines for submitting code & software for further information.

Data

Policy information about availability of data

All manuscripts must include a data availability statement. This statement should provide the following information, where applicable:

- Accession codes, unique identifiers, or web links for publicly available datasets
- A description of any restrictions on data availability
- For clinical datasets or third party data, please ensure that the statement adheres to our policy

The alignment data, inferred phylogenies, simulated data,and time-calibratedd trees are available from Figshare: https://figshare.com/s/a042722630e5ca0b2ba5. 1KP: https://db.cngb.org/onekp/, JGI phycocosm: https://phycocosm.jgi.doe.gov, Phytozome v13: https://phytozome-next.jgi.doe.gov/, NCBI: https://www.ncbi.nlm.nih.gov/. AlgaeBase: https://www.algaebase.org.

Research involving human participants, their data, or biological material

Policy information about studies with human participants or human data. See also policy information about sex, gender (identity/presentation), and sexual orientation and race, ethnicity and racism.

Reporting on sex and gender	N/A
Reporting on race, ethnicity, or other socially relevant groupings	N/A
Population characteristics	N/A
Recruitment	N/A
Ethics oversight	N/A

Note that full information on the approval of the study protocol must also be provided in the manuscript.

Field-specific reporting

Please select the one below that is the best fit for your research. If you are not sure, read the appropriate sections before making your selection.

☐ Life sciences ☐ Behavioural & social sciences ☒ Ecological, evolutionary & environmental sciences

For a reference copy of the document with all sections, see nature.com/documents/nr-reporting-summary-flat.pdf

Ecological, evolutionary & environmental sciences study design

All studies must disclose on these points even when the disclosure is negative.

Study description	Using the most intensive gene sampling of prasinophytes and Prasinodermophyta and coalescent- and concatenation-based approaches to infer: the phylogenetic relationships and the temporal framework of the diversification of green plants.
Research sample	Our research sample contained all classes of Prasinodermophyta, Chlorophyta, Charophyta, and Bryophyta. Our research focuses on the phylogenetic relationship and divergence time of green plants. Therefore, we selected more Viridiplantae sample, and a smallnumber of Glaucophyta,Rhodophyta, and Rhodelphidophyta sample as the outgroups.
Sampling strategy	The basic principle for determining the sample size is: covering all classes of Prasinodermophyta,Chlorophyta, Charophyta, and Bryophyta.Representative species were selected from Glaucophytes,Rhodophytes, and Rhodelphidophytes(Rhodelphidophytes arenot included in the plastid dataset) as the outgroups.
Data collection	The publicly available data was downloaded from the public database by Zhiping Yang using a computer.
Timing and spatial scale	The first public data collection period was 2020.09-2020.12 for four months, and available genomic and transcriptomic data weredownloaded from public databases. The second public data collection period was 2023.05-2023.06 for two months, two genomes ofGlaucophyta, one genome of Rhodophyta, and one transcriptome of Rhodelphidophyta were added to the outgroups for the nucleardataset, as well as two chloroplast genomes of Glaucophyta, and one chloroplast genome of Rhodophyta were added to the outgroups for the plastid dataset. These newly added genomes and transcriptomes were downloaded from public databases.
Data exclusions	No data were excluded.
Reproducibility	To ensure reproducibility of experimental results, we provide the alignment data, and the software and parameters used for eachanalysis are described in detail in the Methods. We consider all experimental results to be reproducible.

Randomization	Bootstrap support for each node in gene trees and species trees was estimated by random resampling of sequence alignment.
Blinding	Blinding is not relevant to our study as we performed phylogenetic/evolutionary analyses of plants.

Did the study involve field work? ☐ Yes ☒ No

Reporting for specific materials, systems and methods

We require information from authors about some types of materials, experimental systems and methods used in many studies. Here, indicate whether each material, system or method listed is relevant to your study. If you are not sure if a list item applies to your research, read the appropriate section before selecting a response.

Materials & experimental systems

n/a	Involved in the study	
☒	☐	Antibodies
☒	☐	Eukaryotic cell lines
☒	☐	Palaeontology and archaeology
☒	☐	Animals and other organisms
☒	☐	Clinical data
☒	☐	Dual use research of concern
☐	☒	Plants

Methods

n/a	Involved in the study	
☒	☐	ChIP-seq
☒	☐	Flow cytometry
☒	☐	MRI-based neuroimaging

Dual use research of concern

Policy information about dual use research of concern

Hazards

Could the accidental, deliberate or reckless misuse of agents or technologies generated in the work, or the application of information presented in the manuscript, pose a threat to:

No	Yes	
☒	☐	Public health
☒	☐	National security
☒	☐	Crops and/or livestock
☒	☐	Ecosystems
☒	☐	Any other significant area

Experiments of concern

Does the work involve any of these experiments of concern:

No	Yes	
☒	☐	Demonstrate how to render a vaccine ineffective
☒	☐	Confer resistance to therapeutically useful antibiotics or antiviral agents
☒	☐	Enhance the virulence of a pathogen or render a nonpathogen virulent
☒	☐	Increase transmissibility of a pathogen
☒	☐	Alter the host range of a pathogen
☒	☐	Enable evasion of diagnostic/detection modalities
☒	☐	Enable the weaponization of a biological agent or toxin
☒	☐	Any other potentially harmful combination of experiments and agents
